# Molecular Characterization of Endoplasmic Reticulum Oxidoreductin 1 from *Bombyx mori*

**DOI:** 10.3390/ijms161125977

**Published:** 2015-11-05

**Authors:** Minchul Seo, Hee-Joo Ryou, Eun-Young Yun, Tae-Won Goo

**Affiliations:** 1 Institute of Medical Research, Dongguk University College of Medicine, Gyeongju 38067, Korea; nansmc@hanmail.net; 2 Department of Agricultural Biology, National Academy of Agricultural Science, RDA, Wanju-gun 55365, Korea; autoomne@gmail.com (H.-J.R.); yuney@korea.kr (E.-Y.Y.); 3 Department of Biochemistry, Dongguk University College of Medicine, Gyeongju 38067, Korea

**Keywords:** *Bombyx mori*, endoplasmic reticulum oxidoreductin 1 (ERO1), protein disulfide isomerase (PDI), disulfide bond

## Abstract

We isolated a complementary DNA (cDNA) clone encoding endoplasmic reticulum oxidoreductin 1 (bERO1, a specific oxidant of protein disulfide isomerase (PDI)) from *Bombyx mori*. This protein has a putative open reading frame (ORF) of 489 amino acids and a predicted size of 57.4 kDa. Although bERO1 protein shares less than 57% amino acid sequence homology with other reported ERO1s, it contains two conserved redox active motifs, a Cys-X-X-X-X-Cys motif of N-terminal and Cys-X-X-Cys-X-X-Cys motif of C-terminal. Both motifs are typically present in ERO1 protein family members. The *bEro1* mRNA expression was highest in posterior silk gland on the sixth day of the 5th instar larvae. Expression of *bEro1* mRNA also markedly increased during endoplasmic reticulum (ER) stress induced by stimulation with antimycin, calcium ionophore A23187, dithiothreitol, H_2_O_2_, monencin, and tunicamycin. In addition, expression levels of *bEro1* exactly coincided with that of *bPdi*. This is the first result suggesting that bERO1 plays an essential role in ER quality control through the combined activities of bERO1 and bPDI as a catalyst of protein folding in the ER and sustaining cellular redox homeostasis.

## 1. Introduction

The endoplasmic reticulum (ER) is a highly specialized organelle involved in the maturation of extracellular membrane proteins and secreted proteins. Disulfide bond formation is a key step in this process [1]. Disulfide bonds are usually formed by pairing and oxidative linkage of sulfhydryl groups (-SH) on cysteine residues during the folding process in the ER. Cooperative activity of two proteins, protein disulfide isomerase (PDI) and flavin adenine dinucleotide (FAD)-dependent oxidase ER oxidoreductin 1 (ERO1) formed disulfide bonds, which both have protein oxidation with redox reactions [2,3]. Two proteins use an exchanging mechanism of thiol-disulfide to transfer disulfide bonds on to their substrate proteins [3]. Disulfide bonds formed between cysteines in the active site of PDI are transferred directly to the folding secretory protein. ERO1 reoxidizes reduced PDI, whereas reduced ERO1 is reoxidized by its FAD cofactor [4,5,6]. The ERO1 contains a conserved Cys-X-X-X-X-Cysmotif (N-terminal) and Cys-X-X-Cys-X-X-Cys motif (C-terminal). The N-terminal Cys-X-X-X-X-Cys motif likely transfers electrons to the two latter residues of the Cys-X-X-Cys-X-X-Cys motif (C-terminal), which are in close proximity to the isoalloxazine ring of FAD [7].

In *Saccharomyces cerevisiae*, most disulfide bonds are formed by thiol-disulfide transfer mechanisms with the oxidized Pdi1p [8,9]. Pdi1p requires other oxidizing molecules to be recycled because Pdi1p is unable to generate disulfide bonds by itself. In previous work, a major disulfide-generating FAD-dependent oxidase Ero1p was identified in yeast [10]. Disulfide bonds are directly transferred to PDI by ERO1 in both yeast and mammalian cells [8]. Frand and Kaiser reported that the oxidative capacity of ER depend on Ero1p activity in *S*. *cerevisiae*. Mutation of Ero1p inhibit yeast resistance to the small-molecule redox reagent DTT and increases the unfolded protein response (UPR) along with secretory protein accumulation. Overexpression of Ero1p improves the oxidizing capacity, as shown by its enhanced resistance to DTT [5,11,12].

Although the functional role and complementary DNA sequence of the ERO1 family of proteins have been demonstrated in a number of eukaryotic organisms and tissues, they have not yet been reported in *B*. *mori*. In this study, we used a genetic approach to investigate regeneration of oxidized PDI catalyzing protein in *B*. *mori*. Here, we present a novel protein termed bERO1, endoplasmic reticulum oxidoreductin 1 of *B*. *mori*, containing two cysteine motifs, N-terminal CAMKYC (Cys-X-X-X-X-Cys motif; active-site cysteine) and C-terminal CVECDKC (Cys-X-X-Cys-X-X-Cys motif; shuttle cysteine). Finally, we provide direct evidence of the gene structure, molecular characterization, and connection between bERO1 and bPDI expression in *B*. *mori* for the first time.

## 2. Results and Discussion

### 2.1. Screening and Analysis of bEro1 cDNA

**Figure 1:**
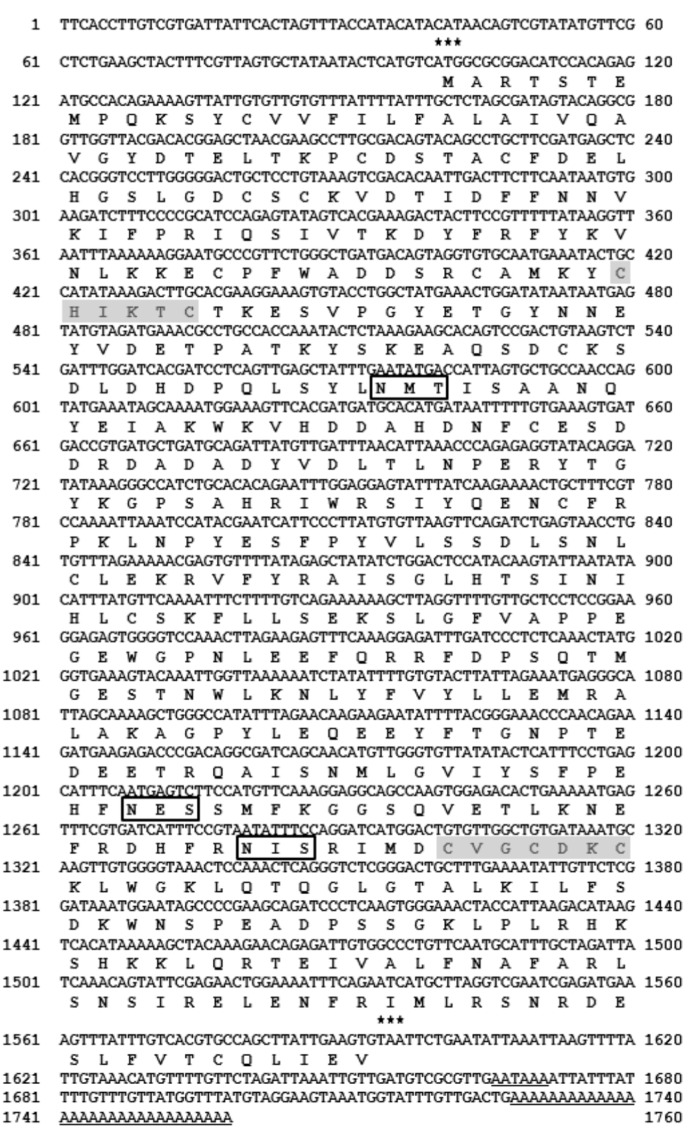
Nucleotide and deduced amino acid sequences of *B. mori* bERO1 cDNA. The predicated amino acid sequence is shown below the nucleotide within the open reading frame. Two activation domains, CHIKTC (111~116 a.a) and CVGCDKC (266~272 a.a), are indicated in the shadow box. The letters in the box indicate three putative glycosylation sites. The translation start and stop codons are indicated by asterisks. The underline nucleotide sequences indicate the putative polyadenylation signal and poly (A) tail, respectively.

To identify novel genes involved in the unfolded protein response in *B*. *mori*–derived Bm5 cells, we used a differential screening method [13]. One of the 768 cDNA clones in the screening shared high homology with the other ERO1 genes, and the cDNA fragment was cloned. Further cDNA sequencing was performed after obtaining a full cDNA using 3′-RACE PCR [14]. This cDNA was shown to contain a putative open reading frame of 489 amino acids and predicted size of 57.4 kDa, whereas the protein shared up to 57% amino acid sequence homology with other reported ERO1s. The gene was named *B*. *mori bEro1* (Table 1), and the *bEro1* sequence was submitted to GenBank under accession number FJ502246. The *bEro1* gene contains a 5′-untranslational region of 99 nucleotides, followed by an initiating ATG codon (Figure 1).

**Table 1:** Comparison of pairwise identifies of *B. mori* endoplasmic reticulum oxidoreductin 1 (*bEro1*) gene and known eukaryotic ERO1 genes.

Scientific Name	1	2	3	4	5	6	7	8	9	10	11	12	13
***B. mori***	**-**												
***A. mellifera***	**57**	**-**											
***T. castaneum***	**57**	**62**	**-**										
***A. aegypti***	**52**	**54**	**61**	**-**									
***D. melanogaster***	**51**	**52**	**54**	**56**	**-**								
***X. tropicalis***	**46**	**44**	**46**	**43**	**45**	**-**							
***M. musculus***	**46**	**45**	**46**	**44**	**45**	**69**	**-**						
***H. sapiens***	**47**	**45**	**48**	**45**	**44**	**57**	**69**	**-**					
***P. toglopytes***	**47**	**45**	**47**	**46**	**45**	**58**	**57**	**58**	**-**				
***B. taurus***	**51**	**50**	**51**	**50**	**48**	**62**	**58**	**58**	**98**	**-**			
***G. gallus***	**48**	**46**	**49**	**46**	**45**	**58**	**62**	**62**	**96**	**96**	**-**		
***D. rerio***	**49**	**47**	**49**	**47**	**47**	**58**	**58**	**60**	**85**	**86**	**92**	**-**	
***S. cerevisiae***	**24**	**28**	**26**	**25**	**26**	**27**	**26**	**29**	**29**	**30**	**27**	**27**	**-**

Sequences were adjusted to optimize alignment of conserved residues, and percentage of aligned identities were determined.

The putative polyadenylation signal (AATAA) is located 70 nucleotides downstream of the termination codon, and the poly A tail is located 57 nucleotides downstream of the putative polyadenylation signal. The bERO1 protein contains two specific regions: an N-terminal CAMKYC (C/X/X/X/X/C motif; active-site cysteine) and C-terminal CVECDKC (Cys-X-X-Cys-X-X-Cys motif; shuttle cysteine) [7]. All eukaryotes contain a shuttle cysteine motif [Cys-V-G(E)-C-D(F,S)-K-Cys] in the C-terminal region of ERO1. However, SIKD amino acid sequences are present between N-terminal cysteines in the active site cysteine motif (Cys-X-X-X-X-Cys) in mammals [15]. In our results, the active site cysteine motif (Cys-X-X-X-X-Cys) in the N-terminus of *B*. *mori* was identical to that in insects [Cys-A-M(I)-R(K)-Y(F)-Cys]. These are marked in gray in Figure 1 and are compared with other known ERO1s in Table 2. In addition, we detected an XBP1-binding site (5′-AACTGACGTGTACTT-3′) in *bEro1* as well as other Ero1 family members (data not shown) [16]. Based on previous cDNA sequence analysis, we propose that *bEro1* gene is a member of the Ero1 gene family.

### 2.2. Tissue Distribution of bEro1 Expression

**Figure 2:**
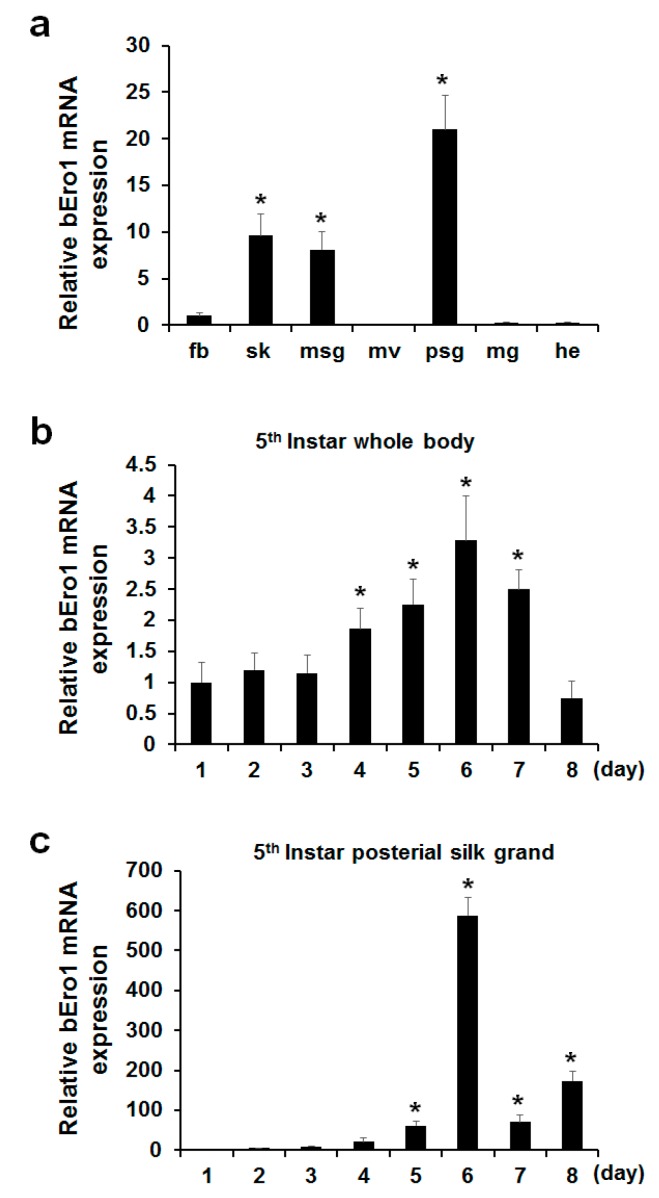
Expression of *bEro1* in various tissues, developmental stages of 5th instar whole body, and posterial silk grand of *B. mori.* (**a**) Expression levels of *bEro1* in various tissues, including fat body (fb), skin (sk), middle silk grand (msg), malpighian vessels (mv), posterial silk grand (psg), midgut (mg) and head (he) in 5th instar three-day larvae. Relative expression levels are presented as the mean ±SD (*n* = 6); * *p* < 0.05 *versus* fat body. Total RNA was extracted from 5th instar (1–8 days) larvae from whole body (**b**) and posterial silk grand (**c**). Relative expression levels are presented as the mean ± SD (*n* = 6); * *p* < 0.05 *versus* day one.

To determine where the *bEro1* gene is expressed in *B. mori*, we isolated fat body (fb), skin (sk), middle silk grand (msg), malpighian vessels (mv), posterior silk grand (psg), midgut (mg), and head (he) from three-day-old 5th instar larvae. After isolation of total RNA, we performed real-time PCR (Figure 2a). Although *bEro1* mRNA was very weakly expressed in fat body, higher expression levels were detected in the posterior silk grand, middle silk grand, and skin. Relative expression levels of the *bEro1* gene were as follows: posterior silk grand > skin > middle silk grand > fat body > midgut > head. However, *bEro1* expression was not detected in the malpighian vessels. Among the tissues tested, highest *bEro1* expression was detected in the posterior silk grand, which showed approximately 21-fold higher *bEro1* expression than fat body. This is the first report demonstrating pronounced expression of an ERO1 family protein (bERO1) in the posterior silk grand of *B. mori*.

### 2.3. bEro1 Expression in Development Stages of 5th Instar Larvae

Silk fibroin consisting of a heavy-chain, light-chain, and P25 is highly expressed in the posterior silk grand in stages of 5th instar larvae compared with other stages to make silk [17]. As ERO1 helps to facilitate protein folding by catalyzing a reaction that forms disulfide bonds, which helps stabilize final protein structures [18], we can assume that *bEro1* was highly expressed at this stage. Thus, we investigated *bEro1* expression in the whole body and posterior silk grand in all development stages of 5th instar larvae. As shown in Figure 2b, *bEro1* was highly expressed in the whole body from four days to seven days in 5th instar larvae. Especially, highest *bEro1* expression was detected after six days in 5th instar larvae, which showed approximately 3.3-fold higher expression compared with day one. Furthermore, *bEro1* was also highly expressed in the posterior silk grand from four days to eight days in 5th instar larvae similar to the whole body (Figure 2c). Especially, highest *bEro1* expression was detected after six days in the posterior silk grand, which showed approximately 588-fold higher expression compared with day one. This result indicates that bERO1 may play an important physiological role related to quality control during synthesis of fibroin in the posterior silk grand.

**Table 2:** Comparison of CXXXXC motifs and CXXCXXC motifs in amino acid sequence of ERO1s.

Scientific Name	N-Terminal (CXXXXC Motif)	C-Terminal (CXXCXXC Motif)
***A. mellifera***	**CHVQPC**	**CVGCDKC**
***T. castaneum***	**CHVEAC**	**CVGCDKC**
***A. aegypti***	**CHVEQC**	**CVGCDKC**
***D. melanogaster***	**CQVENC**	**CVGCDKC**
***X. tropicalis***	**CAVKPC**	**CVGCDKC**
***M. musculus***	**CAVKPC**	**CVGCDKC**
***H. sapiens***	**CHVEPC**	**CVGCDKC**
***P. toglopytes***	**CHVEPC**	**CVGCDKC**
***B. taurus***	**CHVEPC**	**CVGCDKC**
***G. gallus***	**CHVEPC**	**CVGCDKC**
***D. rerio***	**CHVEPC**	**CVGCDKC**
***B. mori***	**CHIKTC**	**CVGCDKC**

### 2.4. Induction of bEro1 during ER Stress

**Figure 3:**
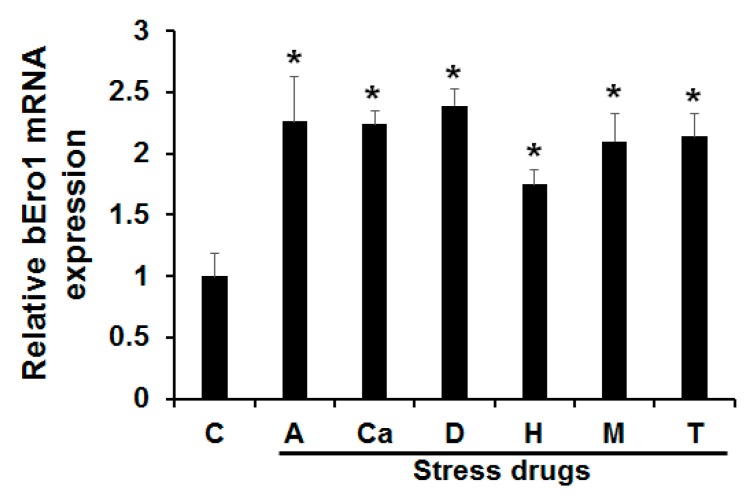
Effects of various endoplasmic reticulum stresses on *bEro1* expression. Bm5 cells were treated with 8 μM antimycin (A), 10 μM calcium inophore A23187 (Ca), 3 mM dithiothreitol (D), 100 μM H_2_O_2_ (H), 100 μM monensin (M), and 5 μg/mL of tunicamycin (T) for 4 h. (C) indicates the control without ER stress reagent. Relative expression levels are presented as the mean ± SD (*n* = 3); * *p* < 0.05 *versus* control.

Cells subjected to ER stress can show drastically altered protein folding, leading to accumulation of unfolded proteins. As a result, this activates a signaling response termed the UPR [19]. A hallmark of this response is coordinated by up-regulation of folding enzymes and ER chaperones. Increased levels of folding enzymes and chaperones during ER stress prevent aggregation of mis- or unfolded proteins and promote proper folding and assembly of proteins in the ER. A previous work demonstrated that Ero1 mRNA levels could be increased in cells subjected to ER stress [20]. In this study, we tested the effects of ER stress on *bEro1* expression. Bm5 cells derived from *B*. *mori* were treated with antimycin A (A), calcium ionphore A23187 (Ci), DTT (D), H_2_O_2_ (H), monensin (M), and tunicamycin (T). As shown in Figure 3, *bEro1* expression was elevated by 2-fold compared with the control. Treatment with DTT, an inhibitor of disulfide bond formation, induced high levels of *bEro1* expression, suggesting *bEro1* may play a role in the formation of disulfide bonds through increased oxidizing capacity, as shown by their increased resistance to reducing agent DTT [8,12]. Furthermore, *bEro1* expression was remarkably increased by treatment with H_2_O_2_. This result indicates that bERO1 plays essential roles in oxidative protein folding and is an oxidizing agent in the ER, similar to ERO1 in mammals [10,11].

### 2.5. Relationship between bERO1 and bPDI

In the case of eukaryote, oxidative protein folding in the ER is an essential function that requires the electron relay system between the proteinaceous components of the pathway [21]. During this process, PDIs directly oxidize new substrate proteins and are subsequently reduced. ERO1 is located upstream of this redox reaction, which reoxidizes and reactivates one of two thioredoxin-like domains of PDI to induce a new cycle of oxidative protein folding through its cofactor FAD [10,12,22]. ERO1 activity plays an essential roles in disulfide bond formation in simple eukaryotes such as yeast and worms. ERO1 function is essential for disulfide bond formation in these types of simple eukaryotes but is largely compensated for by alternative pathways in mammals [23,24]. Although ERO1 participates in alternative mammalian pathways, its importance is highlighted by UPR-mediated up-regulation [21,25]. Formation of disulfide bonds within the ER requires the combined activities of ERO1 and PDI [26].

**Figure 4:**
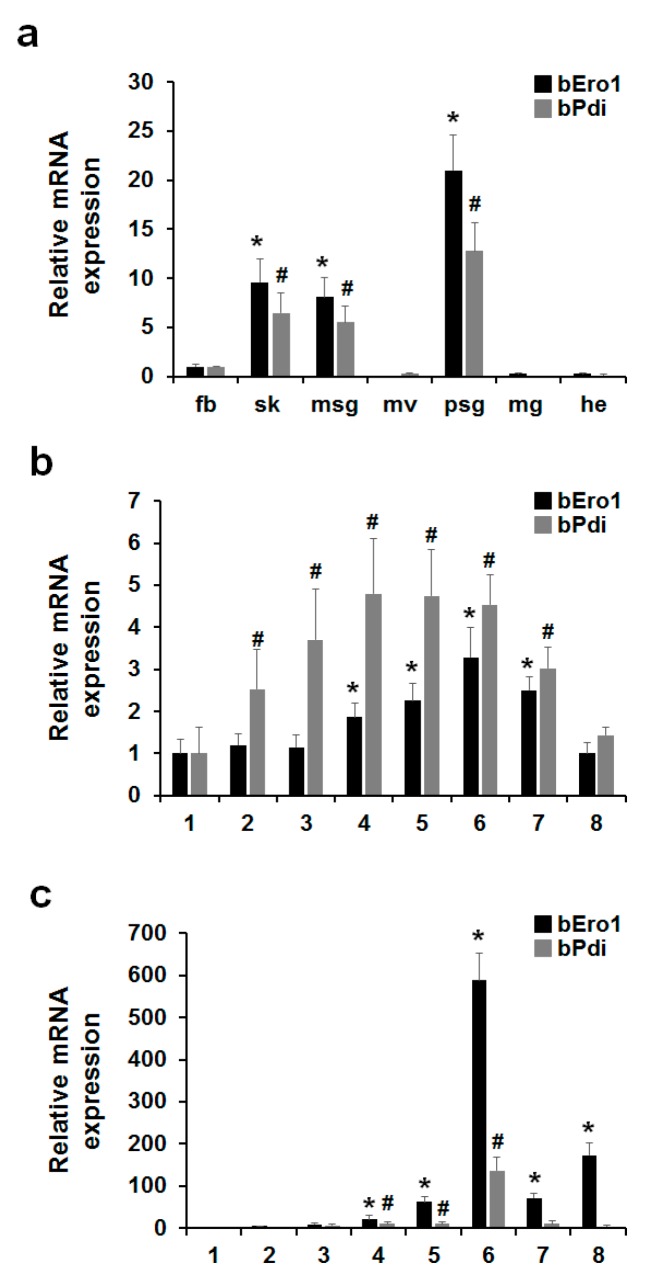
Expression levels of *bEro1* coincide with *bPdi* in *B. mori*. (**a**) Expression levels of *bEro1* and *bPdi* in fat body (fb), skin (sk), middle silk grand (msg), malpighian vessels (mv), posterial silk grand (psg), midgut (mg), and head (he) in three-day-old 5th instar larvae. Relative expression levels are presented as the mean ± SD (*n* = 6); * *p* < 0.05 *versus bEro1* of fat body. ^#^ *p* < 0.05 *versus bPdi* of fat body; (**b**) Expression levels of *bEro1* and *bPdi* in developmental stages of 5th instar whole body (1–8 days). Relative expression levels are presented as the mean ± SD (*n* = 6); * *p* < 0.05 *versus bEro1* on day one. ^#^ *p* < 0.05 *versus bPdi* on day one; (**c**) Expression levels of *bEro1* and *bPdi* in developmental stages of 5th instar posterial silk grand (1–8 days). Relative expression levels are presented as the mean ± SD (*n* = 6); * *p* < 0.05 *versus bEro1* on day one. ^#^ *p* < 0.05 *versus bPdi* on day one.

In this study, we investigated whether or not bPDI and bERO1 are required for oxidative protein folding in the ER as well as mammals. To investigate the relationship between *bPdi* and *bEro1* gene expression in *B*. *mori*, we isolated total RNA from fat body, skin, middle silk grand, malpighian vessels, posterior silk grand, midgut, and head from three-day-old 5th instar larvae, as well as the whole body in all development stages of 5th instar larvae (1–8 days, respectively). As shown in Figure 4, expression of *bEro1* exactly coincided with that of *bPdi* in fat body, skin, middle silk grand, posterior silk grand, and the whole body. Especially, in six-day-old 5th instar larvae, *bPdi* expression drastically increased by about 136-fold compared with the posterior silk grand on day one as well as *bEro1* (588-fold).

A huge amount of silk fibroin is synthesized within cells of the posterior silk grand in silkworm *B*. *mori* in the 5th instar stage. Silk fibroin is composed of H-chain (350 kDa), L-chain (26 kDa), and P25 glycoprotein (30 kDa) [27,28]. H- and L-chains are linked by a disulfide bond between Cys-c20 of the H-chain and Cys-172 of the L-chain, whereas P25 associates with disulfide-linked H- and L-chains via non-covalent interaction [29,30]. This H-L disulfide-linkage is essential for secretion of great amounts of silk fibroin [31]. In our previous work, we reported high expression of bPDI in the posterior silk gland of *B*. *mori* in the 5th instar stage using expressed sequence tag (EST) analysis [32]. The *bEro1* expression also markedly increased in the posterior silk grand of 5th instar larvae as well as *bPdi*. Increasing evidence suggests that ERO1 directly oxidizes PDI, which participates in disulfide bond formation for newly synthesized proteins [3,6,26]. Therefore, cooperative activity and expression of bPDI and bERO1 play important roles in the formation of disulfide linkages among fibroin heavy and light chains in *B*. *mori*.

## 3. Experimental Section

### 3.1. Insects and Cells

The silkworm larvae (Jam 306) were reared on an artificial diet under standard recommended conditions at 24–27 °C, and 70%–90% relative humidity. *B*. *mori* ovary-derived Bm5 cells were maintained at 27 °C in TC-100 insect medium (Sigma, St. Louis, MO, USA) with 10% FBS (fetal bovine serum) (GIBCO Life Technologies, Grand Island, NY, USA) using the standard recommended method [33].

### 3.2. RACE PCR Analysis

To generate the 3′-translated sequence of *bEro1* messenger RNA (mRNA), 3′-RACE PCR was carried out using Bm5 cells, poly (A^+^), and a Marathon cDNA Amplification Kit (Clontech, Palo Alto, CA, USA) according to the manufacturer’s protocol. The adapter sequence attached to the ends of the cDNA allowed its usage in 5′- and 3′-RACE. Three GSPs (gene specific primers) were designed for detecting 3′-RACE fragment of the *bEro1* cDNA. Nucleotide sequences of the primer set: GSP1, 5′-GCTCCTCCGGAAGGAGAGT-GGGTC-3′; GSP2, 5′-CCGACAGGCGATCAGCAACATGTTG-3′; GSP3, 5′-GGGTCTCGGGAC-TGCTTTGAAAATATTGTTCT-3′. These primers were used in 3′-RACE in conjunction with the adaptor primer (AP1,5′-CCATCCTAATACGACTCACTATAGGGC-3′) to amplify the 3′-ends of the gene from cDNA. PCR conditions were as follows: initial denaturation at 94 °C for 3 min, then 30 cycles of 94 °C for 30 s, 60 °C for 30 s, and 72 °C for 1 min followed by 72 °C for 10 min. PCR products were subcloned using pGEM-T Easy Vector (Promega, WI, USA), and subcloned cDNA fragment were then sequenced.

### 3.3. Reverse Transcription PCR

Quantitative real-time PCR was carried out using the total cellular RNA extracted using a Total RNA Isolation Kit (Promega, Madison, WI, USA) according to the manufacturer’s protocol. PCR was performed using GSP set at an annealing temperature of 60 °C for 40 cycles using a SYBR Premix Ex Tag (Takara, Shiga, Japan). Nucleotide sequences of the primer set: actin3 forward primer, GAAGCTGTGCTACGTCGCTC; actin3 reverse primer, CCGATGGTGATGACCTGACC; *bEro1* forward primer, CCATTAGTGCTGCCA-ACCAGTA; *bEro1* reverse primer, ATCTGCATCAGCATCACGGTC; *bPdi* forward primer, CTAGCGAAAGTTGACGCAACTC; *bPdi* reverse primer, TGCATAGGACTGCCATTCCTG [34]. Actin3 was used as an internal control.

### 3.4. Statistical Analysis

All data are presented as the mean ± S.D. from three or more independent experiments, unless otherwise stated. Different treatments were compared with Student’s *t*-test, one-way ANOVA with Dunnett’s multiple comparisons test, or chi-square tests using SPSS software (version 18.0; SPSS Inc., Chicago, IL, USA). Differences with a *p*-value less than 0.05 were considered to be statistically significant.

## 4. Conclusions

In conclusion, this result suggests that disulfide bond formation within the ER requires combined expression of bERO1 and bPDI in *B*. *mori* as well as yeast and mammals. However, further studies are warranted to clarify the role of bERO1 in reoxidizing bPDI for disulfide bond formation and maintaining an oxidative environment in the ER through gain- or loss-of-function studies.
